# *OPA1* and disease-causing mutants perturb mitochondrial nucleoid distribution

**DOI:** 10.1038/s41419-024-07165-9

**Published:** 2024-11-30

**Authors:** J. Macuada, I. Molina-Riquelme, G. Vidal, N. Pérez-Bravo, C. Vásquez-Trincado, G. Aedo, D. Lagos, P. Yu-Wai-Man, R. Horvath, T. J. Rudge, B. Cartes-Saavedra, V. Eisner

**Affiliations:** 1 Facultad de Ciencias Biológicas, Pontificia Universidad Católica de Chile, Santiago, Chile; 2 Institute for Biological and Medical Engineering, Schools of Engineering, Biology, and Medicine, Pontificia Universidad Católica de Chile, Santiago, Chile; 3 Interdisciplinary Computing and Complex Biosystems (ICOS) research group, School of Computing, Newcastle University, Newcastle upon Tyne, UK; 4 Department of Clinical Neurosciences, John Van Geest Centre for Brain Repair, University of Cambridge, Cambridge, UK; 5 Cambridge Eye Unit, Addenbrooke’s Hospital, Cambridge University Hospitals, Cambridge, UK; 6 Moorfields Eye Hospital NHS Foundation Trust, London, UK; 7 Institute of Ophthalmology, University College London, London, UK; 8 Department of Chemical and Bioprocess Engineering, School of Engineering, Pontificia Universidad Católica de Chile, Santiago, Chile; 9 MitoCare Center for Mitochondrial Imaging Research and Diagnostics, Department of Pathology and Genomic Medicine, Thomas Jefferson University, Philadelphia, PA USA

**Keywords:** Energy metabolism, Super-resolution microscopy

## Abstract

Optic atrophy protein 1 (OPA1) mediates inner mitochondrial membrane (IMM) fusion and cristae organization. Mutations in OPA1 cause autosomal dominant optic atrophy (ADOA), a leading cause of blindness. Cells from ADOA patients show impaired mitochondrial fusion, cristae structure, bioenergetic function, and mitochondrial DNA (mtDNA) integrity. The mtDNA encodes electron transport chain subunits and is packaged into nucleoids spread within the mitochondrial population. Nucleoids interact with the IMM, and their distribution is tightly linked to mitochondrial fusion and cristae shaping. Yet, little is known about the physio-pathological relevance of nucleoid distribution. We studied the effect of OPA1 and ADOA-associated mutants on nucleoid distribution using high-resolution confocal microscopy. We applied a novel model incorporating the mitochondrial context, separating nucleoid distribution into the array in the mitochondrial population and intramitochondrial longitudinal distribution. Opa1-null cells showed decreased mtDNA levels and nucleoid abundance. Also, loss of Opa1 led to an altered distribution of nucleoids in the mitochondrial population, loss of cristae periodicity, and altered nucleoids to cristae proximity partly rescued by OPA1 isoform 1. Overexpression of WT OPA1 or ADOA-causing mutants c.870+5 G > A or c.2713 C > T in WT cells, showed perturbed nucleoid array in the mitochondria population associated with cristae disorganization, which was partly reproduced in Skeletal muscle-derived fibroblasts from ADOA patients harboring the same mutants. Opa1-null and cells overexpressing ADOA mutants accumulated mitochondria without nucleoids. Interestingly, intramitochondrial nucleoid distribution was only altered in Opa1-null cells. Altogether, our results highlight the relevance of OPA1 in nucleoid distribution in the mitochondrial landscape and at a single-organelle level and shed light on new components of ADOA etiology.

## Introduction

Mitochondria are critical to intracellular bioenergetic balance [1] and their genome, the mtDNA, encodes genes required to maintain the function of the electron transport chain (ETC). MtDNA maintenance defects are a hallmark of bioenergetic dysfunction in several diseases and aged tissue [2, 3]. In humans and mice, mtDNA is packaged into 70–110 nm ellipsoid DNA-protein complexes named nucleoids [4, 5] through interaction with its main protein component, the Mitochondrial Transcription Factor A (TFAM) [6, 7].

The maintenance and distribution of mtDNA are critical for cellular physiology [8–13] and are impaired during disease [14–19]. Mitochondria undergo constant fusion/fission cycles, which allow the exchange of its components and supports mtDNA maintenance [1, 8, 20]. In mammalian cells, nucleoids are semi-regularly distributed inside the mitochondrial population [5, 21, 22] and, in yeast, it was proven that regular distribution is necessary to accurately control nucleoid segregation to daughter cells [23]. Furthermore, nucleoid positioning on the sites of mitochondrial fission can ensure mtDNA inheritance to daughter mitochondria [24]. Regardless of the relevance of mtDNA abundance and maintenance, little is known about the physio-pathological relevance of nucleoid distribution in the mammalian mitochondrial population, partly because of the challenge of studying nanoscopic structures [25].

Nucleoids physically interact with the inner mitochondrial membrane (IMM), and are interspersed between the IMM cristae [4, 26, 27]. Cristae structure is tightly regulated by multiple proteins, such as the ATP synthase [28], the Mitochondrial Contact Site and Cristae Organization System (MICOS) [29, 30], and OPA1 [31–33]. OPA1 is a GTPase also involved in various processes, including mitochondrial fusion and fission [34–36]. Furthermore, the lack of Mgm1, the OPA1 yeast ortholog, leads to mtDNA depletion [37, 38] and *Opa1*^−/−^ cells show a decrease in mtDNA and nucleoid number [39–41], and display mitochondria without nucleoids [39]. Finally, OPA1exon4b isoforms 3, 5, 6, and 8 physically interact with mtDNA to regulate its maintenance [39, 42]. Yet, the role of OPA1 in intramitochondrial nucleoid distribution in mammalian cells under pathophysiological conditions is underexplored.

*OPA1* mutations lead to Autosomal Dominant Optic Atrophy (ADOA, MIM 165500), a disease that causes progressive loss of retinal ganglion cells resulting in optic nerve degeneration and blindness [43]. About 20% of ADOA patients manifest a more severe syndromic form of the disease, classified as ADOA plus (ADOA + , MIM: 125250) [44, 45]. Patients bearing mutations at the GTPase domain of OPA1 are more likely to develop ADOA+ [45]. Skeletal muscle samples from ADOA patients display defects in mtDNA integrity and distribution with bioenergetic dysfunction markers, as well as IMM ultrastructure alterations [18, 46, 47]. Accordingly, our previous study showed that OPA1 ADOA-causing mutants in the GTPase or GTPase effector domain (GED) display distinct effects on IMM ultrastructure and mitochondrial fusion and fission dynamics [48]. Nevertheless, it is unclear if these changes have an impact on mtDNA distribution and maintenance.

In this work we studied the effect of OPA1 and different ADOA-causing mutants on nucleoid distribution. We used *Opa1*^−/−^ MEF and overexpression of OPA1 mutants on WT MEF along with patient-derived cells to study nucleoid distribution and nucleoid-cristae proximity. Our data showed that the lack of OPA1 and the overexpression of OPA1 or ADOA-causing mutants perturbed nucleoid cluster distribution within the mitochondrial population and disrupted cristae morphology. Furthermore, nucleoid-cristae proximity is perturbed in *Opa1*^−/−^ cells which might contribute to local OXPHOS dysfunction. This work introduces new clues on the influence of OPA1 over mitochondrial nucleoid distribution and provides novel insights on the etiology of the mtDNA alterations as well as the local bioenergetic dysfunction found in ADOA patients.

## Results

### Opa1 determines nucleoid abundance and distribution

**Fig. 1:**
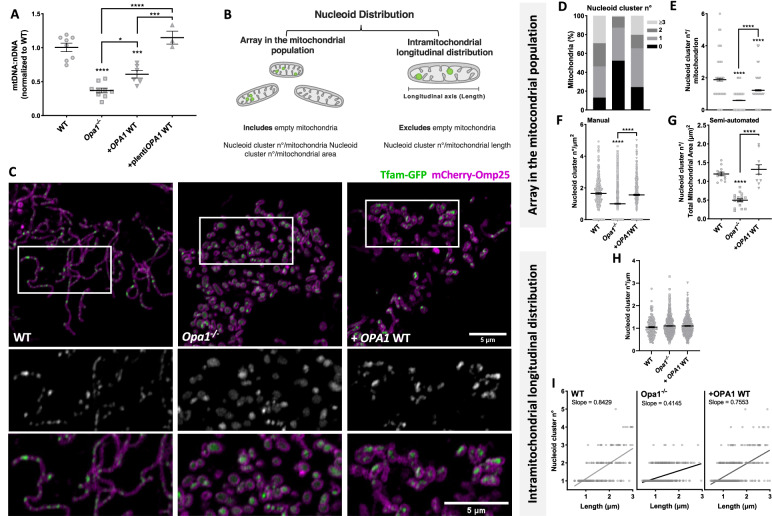
Opa1 determines nucleoid abundance and array in the mitochondrial population and intramitochondrial longitudinal distribution. **A** Mitochondrial DNA abundance in WT MEF, *Opa1*^−/−^ and *Opa1*^−/−^ cells stably expressing a lentiviral plasmid carrying OPA1 cDNA, quantified by qPCR and expressed as mt-Nd4 to Gapdh ratio. Data are mean ± SEM of ≥ 3 independent experiments. **B** Schematic representation of the components of nucleoid distribution. **C** Representative images of WT MEF, *Opa1*^−/−^ and *Opa1*^−/−^ cells with WT OPA1 acute expression, co-transfected with mCherry-Omp25 (OMM) and Tfam-GFP (nucleoids) cDNA. Bottom panel, insets of Tfam-GFP (top) and merge (bottom). **D** Percentage of mitochondria with different number of nucleoid clusters. **E** Mean nucleoid cluster number per mitochondrion. **F** Mean nucleoid cluster number per mitochondrion area, manually quantified. **G** Number of nucleoid clusters per total mitochondrial area, semi-automatically quantified by the MiNuD algorithm. For *Opa1*^−/−^ cells, DNA was labeled using PicoGreen, and mitochondria were labeled with mCherry-Omp25 (OMM) or MitoTracker Deep Red. **H** Mean nucleoid cluster number per mitochondrion length. **I** Simple regression of the relationship between nucleoid cluster number and mitochondrion length in individual organelles. Data are mean ± SEM of ≥ 281 objects from ≥ 15 cells of ≥ 3 independent experiments, and for G data are mean ± SEM of ≥ 10 cells of ≥ 2 independent experiments (****p < 0.0001; ***p < 0.0005; *p < 0.05).

We studied the role of OPA1 in nucleoid abundance using *Opa1*^−/−^ MEF and rescue through expression of human OPA1 isoform 1. First, we quantified the mtDNA copy number and found a ~ 70% decrease in mtDNA levels in *Opa1*^−/−^ cells, which was not explained by changes in mitochondrial biogenesis transcriptional regulator PCG1-α [1] (Fig. S1, Supplementary material), or mitochondrial protein mass decrease, as we previously reported [48]. The mtDNA levels were partially rescued upon acute expression of OPA1, and completely rescued in our Opa1^−/−^ + OPA1 stable cell line ( + p-lenti OPA1 WT) (Fig. 1A). To accurately evaluate nucleoid distribution, we propose a novel model where we measure: (1) the nucleoids array in the mitochondrial population, which describes the density and spread of nucleoids within the mitochondrial network and includes the mitochondria with and without nucleoids and, (2) intramitochondrial longitudinal distribution, which describes nucleoid localization in relation to the length of single organelles (Fig. 1B). To apply our model, we developed a manual approach and a novel unbiased semi-automated method called MiNuD (Mitochondrial Nucleoids Distribution), as described in Methods (Fig. S2).

We applied our semi-automatic approach to quantify nucleoid distribution in WT MEF using two strategies to label nucleoids: (i) direct dsDNA labeling with PicoGreen or (ii) Tfam-GFP [49]. We performed live-cell imaging at a high-resolution confocal microscope, with sufficient resolution to identify nucleoid clusters, rather than individual objects [50]. Both techniques showed “point-like” signals with apparent uniform distribution throughout the mitochondrial network (Fig. S3A), and comparable number of nucleoid clusters per total mitochondrial area (Fig. S3B). Considering that PicoGreen displayed a nuclear stain (Fig. S3A), we continued our study with Tfam-GFP, excluding cells that show signs of major overexpression such as nuclear Tfam-GFP localization [49].

We evaluated mitochondrial nucleoid abundance and distribution using the manual quantification approach described in Methods (Fig. S2). We studied the number of nucleoids per mitochondria in maximum projections ( ~ 1 µm thickness) and found that in WT MEF, 13% of mitochondria lack apparent Tfam-GFP foci (Fig. 1C, D), thus we classified them as “empty”. We corroborated this result with anti-dsDNA and Picogreen staining (Fig. S3C).

*Opa1*^−/−^ cells displayed an increase in “empty” organelles (52% vs 13% WT) and a diminished number of nucleoid clusters per mitochondria, consistent with the loss in mtDNA levels (Fig. 1C, D). Acute rescue of OPA1 partially recovered the number of “empty” mitochondria (24%) and the number of nucleoid clusters per mitochondria (Fig. 1C–E). In addition, we measured the number of nucleoid clusters per mitochondrial area using our manual approach and MiNuD, and found the same tendency (Fig. 1F–G). Notably, in *Opa1*^−/−^ cells, Tfam-GFP showed a combination of bright points and diffuse signals (Fig. 1C). The acute rescue of OPA1 re-established the nucleoid cluster phenotype and labeling. Alternative nucleoid labeling with PicoGreen or anti-dsDNA confirmed the low number of nucleoid clusters observed in the *Opa1*^−/−^ cells (Fig. S4A–C).

Considering the high number of mitochondria lacking apparent Tfam-GFP foci and the changes in morphology shown in *Opa1*^−/−^ cells, we evaluated intramitochondrial longitudinal distribution in organelles that: (1) showed Tfam-GFP foci; (2) were shorter than 3 µm, the longest organelles found in *Opa1*^−/−^ cells (Fig. S5). Interestingly, the average intramitochondrial distribution of one nucleoid cluster per micrometer remained unchanged in *Opa1*^−/−^ cells (Fig. 1H). To further examine this, we applied a simple linear regression to the number of nucleoid clusters found in each mitochondrion and its length. WT MEF showed a correlation between mitochondria length and nucleoid cluster number that is reduced in *Opa1*^−/−^ cells and rescued after acute OPA1 expression (Fig. 1I).

Thus, OPA1 acute rescue in *Opa1*^−/−^ cells partially recovered mtDNA abundance and the nucleoid cluster array in the overall mitochondrial population. Notably, the absence of OPA1 perturbed the intramitochondrial longitudinal distribution of the nucleoid clusters.

### OPA1 rescues mitochondrial cristae periodicity and nucleoid-cristae proximity in *Opa1*^−/−^ cells

**Fig. 2:**
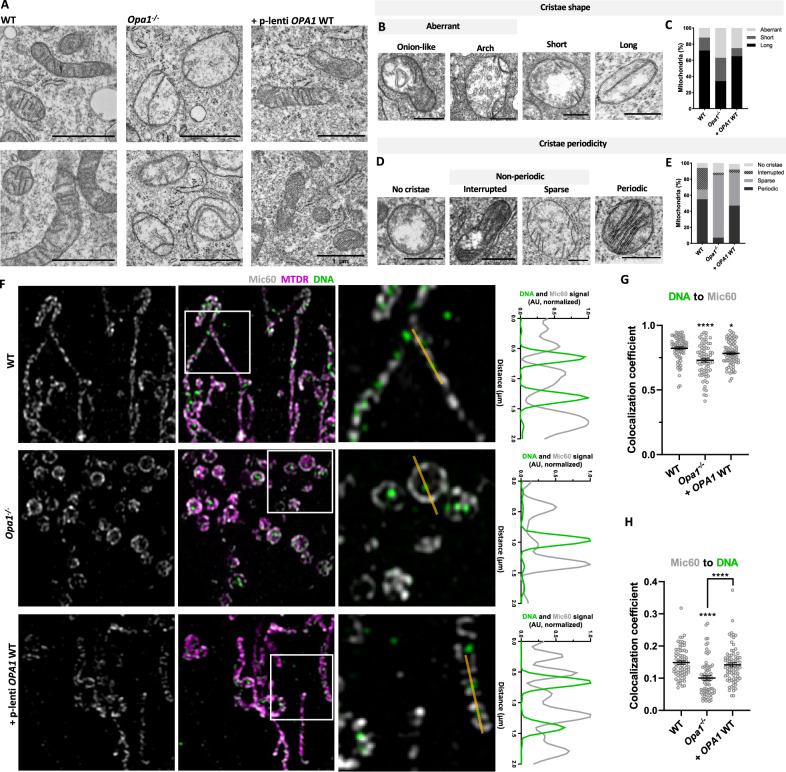
The stable expression of WT OPA1 rescues mitochondrial cristae periodicity and nucleoid-cristae proximity in *Opa1*^−/−^ cells. **A** Representative TEM images of WT MEF, *Opa1*^−/−^ and *Opa1*^−/−^ cells stably expressing a lentiviral plasmid carrying OPA1 cDNA. **B** Mitochondrial cristae shape classification and **C** their quantification**. D** Mitochondrial cristae periodicity classification and **E** their quantification. Data are from ≥ 98 objects from ≥ 2 independent experiments. **F** Representative immunofluorescence images of WT MEF, *Opa1*^−/−^ and *Opa1*^−/−^ cells stably expressing a lentiviral plasmid carrying OPA1 cDNA. Cells were labeled with MitoTracker Deep Red and antibodies against dsDNA and Mic60. The white boxes correspond to 5 x 5 μm insets. (Right) Intensity profile of dsDNA and Mic60 signals along the yellow line from left to right (2 µm). **G**, **H** Colocalization coefficients for dsDNA and Mic60 in the cells shown in F. Each point represents one cell. Data are mean ± SEM from ≥ 70 cells from 4 independent experiments (****p < 0.0001; *p < 0.05). In A, B and D the scale bar is 1 μm.

As OPA1 is key for IMM fusion and cristae organization [32, 40] and the cristae morphology is associated with nucleoid distribution and abundance [10, 26, 41], we performed transmission electron microscopy (TEM) (Fig. 2A). Based on the diversity of cristae phenotypes, we classified each mitochondrion according to their cristae shape and periodicity (Fig. 2B–D). Mitochondria with no discernible cristae were classified as organelles with “no cristae”.

In *Opa1*^−/−^ cells, we observed an increase in mitochondria with short (29% vs. 16% WT) and aberrant cristae (37% vs. 12% WT) (Fig. 2A–C). The aberrant cristae in *Opa1*^−/−^ cells consisted mainly of onion-like and arch phenotypes (Fig. S4D). Importantly, the stable expression of OPA1 rescued the abundance of long cristae (65% vs. 34% *Opa1*^−/−^) (Fig. 2A–C). Similarly, cristae periodicity was disturbed in *Opa1*^−/−^ cells. Mitochondria with no cristae were increased (12% vs. 6% WT) as well as non-periodic sparse cristae (78% vs. 12% WT) (Fig. 2E). The stable expression of OPA1 partially rescued the abundance of periodic cristae (47% vs. 7% *Opa1*^−/−^) (Fig. 2E).

Considering the major changes in IMM ultrastructure in *Opa1*^−/−^ cells, we next looked at the nucleoid-cristae proximity, by immunofluorescence labeling of dsDNA and MICOS component Mic60. In WT MEF, the nucleoids are abundant and interspersed between the cristae as expected, displaying close mutual proximity (Fig. 2F). However, in *Opa1*^−/−^ cells, there is an apparent disorganization of the nucleoid-cristae proximity. These data were further evaluated by analyzing the colocalization of mtDNA to Mic60 and vice versa. We found that mtDNA colocalization to Mic60 is decreased in *Opa1*^−/−^ cells and is mildly rescued after OPA1 stable expression, hence, nucleoid-cristae proximity is perturbed in mitochondria that contain nucleoids (Fig. 2F–G). In contrast, Mic60 colocalization to mtDNA is rescued in *Opa1*^−/−^ cells after OPA1 expression, which relates to the mtDNA content and cristae periodicity recovery (Fig. 2F, H).

Thus, in *Opa1*^−/−^ cells, mitochondria predominantly display sparse cristae with decreased nucleoid-cristae proximity, that are partly rescued by OPA1 stable expression.

### Acute expression of WT and pathogenic *OPA1* variants perturb mitochondrial nucleoid abundance and distribution

**Fig. 3:**
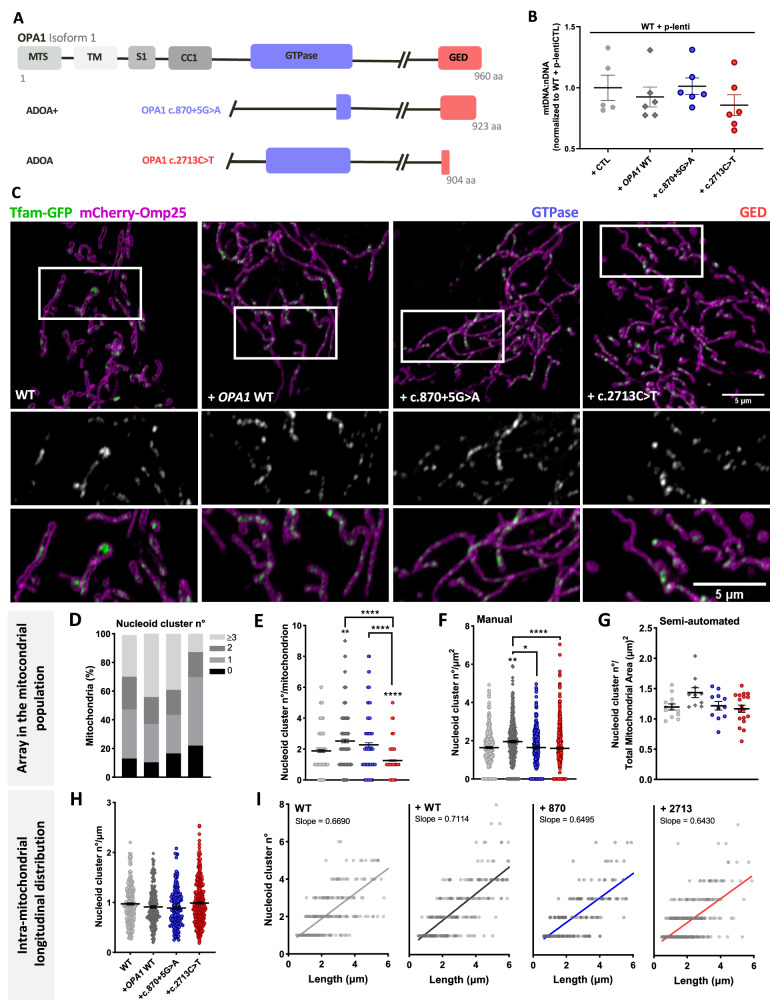
Acute expression of WT and pathogenic *OPA1* variants perturb mitochondrial nucleoid cluster abundance and distribution. **A** Schematic representation of OPA1 isoform 1 domains and ADOA-causing mutants: OPA1 c.870+5 G > A (GTPase domain) and OPA1 c.2713 C > T (GTPase effector domain, GED). MTS: mitochondrial targeting sequence. TM: transmembrane domain. S1: Processing site 1. CC1: coiled-coil domain 1. **B** Mitochondrial DNA abundance of WT MEF stably expressing a lentiviral plasmid carrying control cDNA (CTL), *OPA1* WT, or pathogenic *OPA1* variants; quantified by qPCR and expressed as mt-Nd4 to Gadph ratio. Data are mean ± SEM from ≥ 5 independent experiments. **C** Representative images of WT MEF, WT MEF overexpressing *OPA1* WT, or pathogenic *OPA1* variants, co-transfected with mCherry-Omp25 (OMM) and Tfam-GFP (nucleoids) cDNA. Bottom panel, insets of Tfam-GFP (top), and merge (bottom). **D** Percentage of mitochondria with a different number of nucleoid clusters. **E** Mean nucleoid cluster number per mitochondrion. **F** Mean nucleoid cluster number per mitochondrion area, manually quantified. **G** Mean nucleoid cluster number per total mitochondrial area, quantified semi-automatically by the MiNuD algorithm. **H** Mean nucleoid cluster number per mitochondrion length. **I** A simple regression of the relation between mitochondrial length and the number of nucleoids in single organelles. Data are mean ± SEM of ≥ 228 objects from ≥ 18 cells of ≥ 4 independent experiments. For **G**, data are mean ± SEM of ≥ 11 cells of ≥ 4 independent experiments (****p < 0.0001; **p < 0.005; *p < 0.05).

We next inspected the effect of OPA1 ADOA-causing mutants on nucleoid distribution and abundance to further understand what causes the mtDNA defects found in ADOA patients [18, 19, 46, 47]. We used our previously characterized model of overexpression of OPA1 in WT MEF and expression of two OPA1 ADOA-causing mutants [48, 51]: *OPA1* c.870+5 G > A (p. Lys262_Arg290Del), located in the GTPase domain; and c.2713 C > T (p.Arg905X), located in the GTPase-effector domain (GED) (Fig. 3A).

We first analyzed mtDNA maintenance. Interestingly, acute, and stable overexpression of OPA1 showed no differences in mtDNA copy number (Fig. S6A, Fig. 3B). Likewise, mtDNA abundance was unchanged after stable overexpression of OPA1 mutants (Fig. 3B) or TFAM overexpression (Fig. S6B). Moreover, the stable expression of OPA1 mutants did not cause mtDNA deletions (Fig. S6C–D) or changes in PCG1-α levels (Fig. S1).

The nucleoid array showed that acute OPA1 overexpression caused a slight reduction in empty mitochondria (10% vs. 13% WT MEF) and an increase in the number of nucleoid clusters per mitochondrion and per mitochondrion area (Fig. 3C–G). Conversely, cells with OPA1 c.870+5 G > A expression showed an increase in “empty” mitochondria (17% vs. 10% WT OPA1) and a lower abundance of nucleoids clusters per mitochondrion area (Fig. 3C–G). Furthermore, cells expressing the mutant OPA1 c.2713 C > T showed an increase in “empty” mitochondria (22% vs. 10% WT OPA1) and a reduction in the number of nucleoid clusters per mitochondrion and area (Fig. 3C–G). These changes were independent of the mitochondrial morphology since WT OPA1 and c.870+5 G > A mutant overexpression showed elongated mitochondria, and cells expressing OPA1 c.2713 C > T exhibited fragmented mitochondria (Fig. S7A–D), as we previously described in this model [48]. Intramitochondrial longitudinal distribution of nucleoids was quantified in mitochondria up to 6 µm, the longest organelles found in OPA1 c.2713 C > T (Fig. S7C). Interestingly, we found no changes in this parameter upon the expression of WT OPA1 or the studied mutants (Fig. 3H–I).

Thus, in our model neither OPA1 overexpression nor the expression of ADOA-causing mutants induced changes in mtDNA abundance and integrity. Yet, OPA1 overexpression caused mitochondrial elongation and an increased nucleoid cluster abundance in the mitochondrial population. Surprisingly, cells expressing OPA1 ADOA-causing mutants showed alterations in mitochondrial morphology and nucleoid array in the mitochondrial population without altering the intramitochondrial longitudinal distribution of the nucleoids. Hence, this heterozygous-like system constitutes a pathologically relevant model to study nucleoid distribution upon alterations of mitochondrial dynamics without mtDNA depletion.

### Cristae ultrastructure and nucleoid-cristae proximity in cells expressing *OPA1* ADOA-causing mutants

**Fig. 4:**
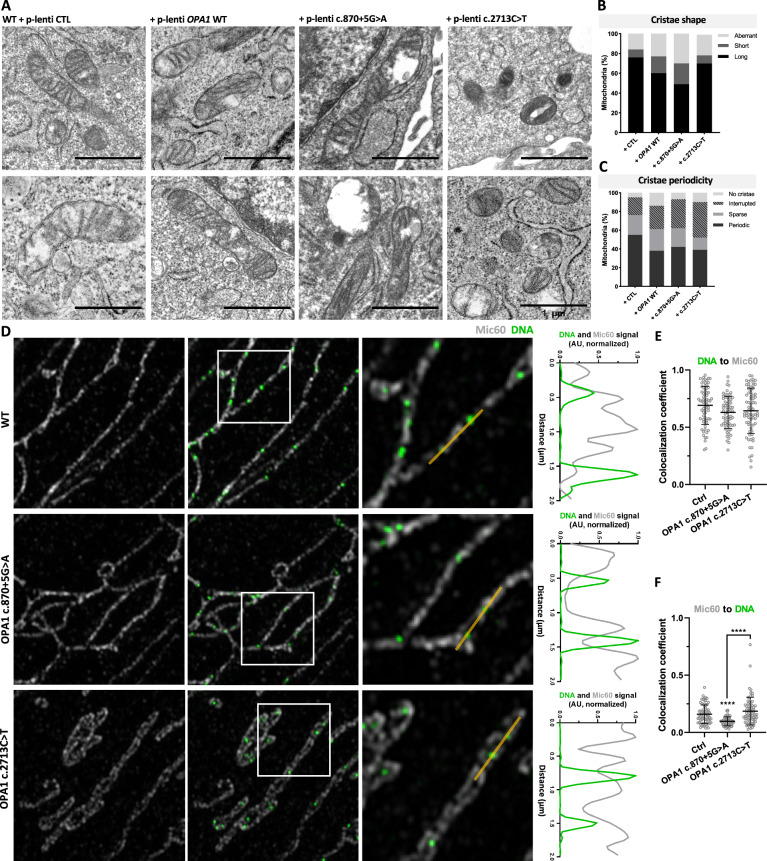
Cristae ultrastructure and nucleoid-cristae proximity in cells expressing *OPA1* ADOA-causing mutants. **A** Representative TEM images of WT MEF stably expressing a lentiviral plasmid containing control cDNA (CTL), *OPA1* WT, or pathogenic *OPA1* variants. Scale bar is 1 μm. **B** Quantification of mitochondrial cristae shape descriptors. **C** Quantification of mitochondrial cristae periodicity. Data are from ≥ 112 objects from ≥ 3 independent experiments. **D** Representative immunofluorescence images of Skeletal muscle-derived fibroblasts from ADOA patients carrying OPA1 variants. Cells were labeled with antibodies against dsDNA and Mic60. The white boxes correspond to the 5 x 5 μm insets. (Right) Intensity profile of dsDNA and Mic60 signals along the yellow line from left to right (2 µm). **E**, **F** Colocalization coefficients for dsDNA and Mic60 in the cells shown in D. Each point represents one cell. Data are mean ± SEM from ≥ 70 cells from 4 independent experiments (****p < 0.0001).

Previous data indicate that ADOA patients show IMM cristae defects [40, 48, 52]. Thus, we further evaluated the IMM ultrastructure. Our data showed that stable OPA1 overexpression leads to an increase in short cristae (17% vs. 8% CTL) and aberrant cristae (23% vs. 16% CTL) (Fig. 4A–B). OPA1 c.870+5 G > A mutant’s expression caused the same tendency, with an increase in short cristae (21% vs. 17% WT OPA1) and aberrant cristae (30% vs. 23% WT OPA1). Interestingly, cells expressing the mutant c.2713 C > T showed similar abundance of aberrant cristae (21% vs. 23% WT OPA1) but a decrease in short cristae (8% vs. 17% WT OPA1) (Fig. 4A–B).

We then quantified cristae periodicity and found that cells with stable overexpression of OPA1 showed a decrease in periodic cristae (Fig. 4A–C). The expression of both mutants caused an increase in the interrupted cristae phenotype (31% c.870+5 G > A; 38% c.2713 C > T vs. 25% WT OPA1) (Fig. 4A–C).

We further explored if the cristae defects impact nucleoid-cristae proximity in Skeletal muscle-derived fibroblasts of ADOA patients. In all the conditions, nucleoids seemed in close proximity to the cristae, which was corroborated by the mtDNA to Mic60 colocalization (Fig. 4D–E). However, the Mic60 colocalization to mtDNA was decreased in the cells carrying the mutant c.870+5 G > A (Fig. 4F), which might be explained by the increased aberrant cristae present in these cells (Fig. 4B).

Therefore, OPA1 is relevant for cristae structuration and nucleoid array in the mitochondrial population. Nevertheless, we did not observe major domain-specific differences of OPA1 mutants on nucleoid distribution, IMM structure, and nucleoids-to-cristae proximity.

## Discussion

**Fig. 5:**
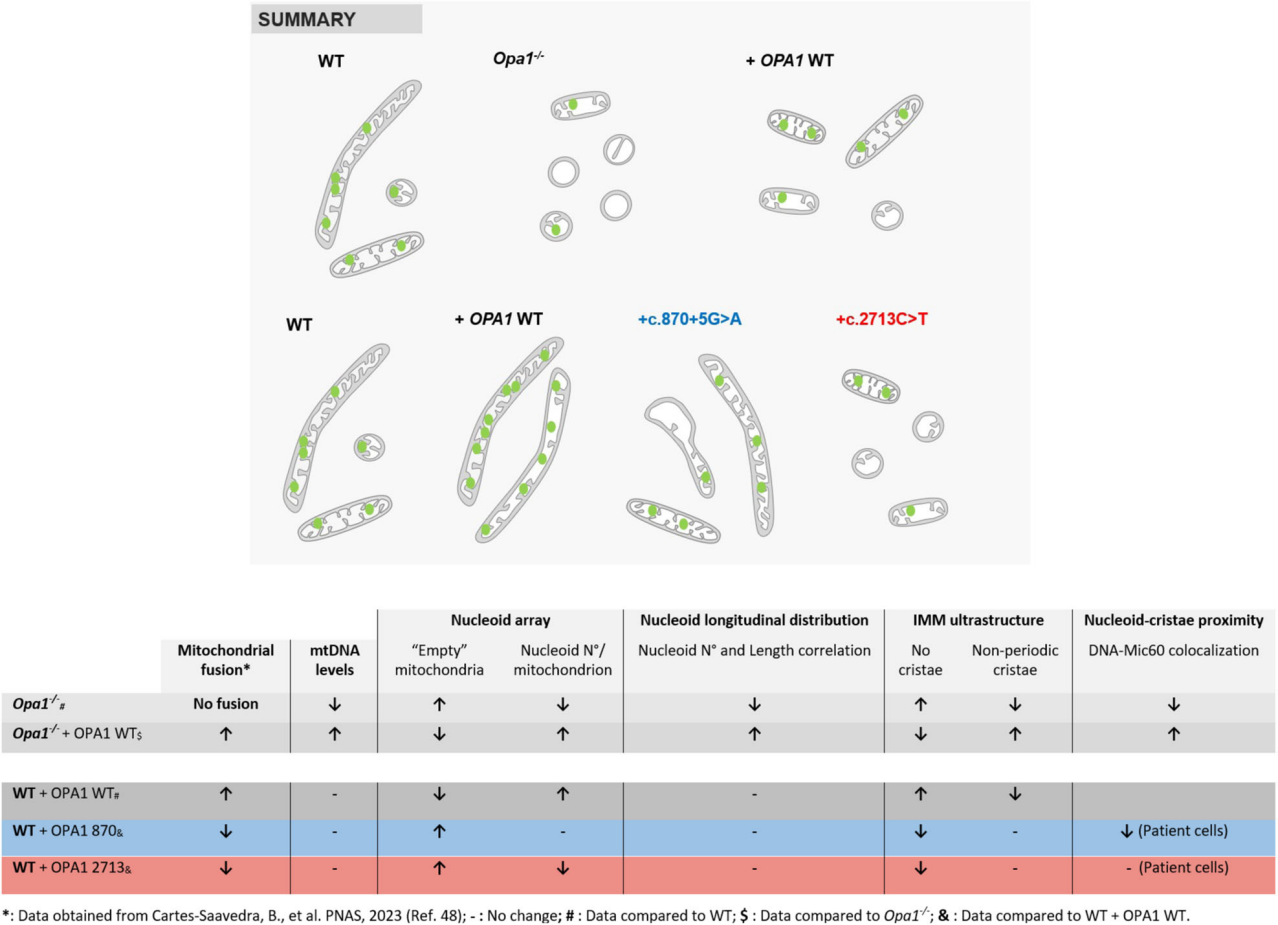
Summary of main findings. The cartoon and table show mitochondrial morphology and cristae ultrastructure changes, as well as nucleoid (green circles) abundance and nucleoid distribution alterations in the studied models. Mitochondrial fusion data were obtained from Cartes-Saavedra, et al. [48].

Here, we demonstrated that the absence of OPA1 or the expression of OPA1 ADOA-causing mutants, lead to an altered nucleoid array, suggesting that the execution of mitochondrial fusion is needed to keep nucleoid distribution amongst the mitochondrial population. Also, single-organelle analysis showed that the absence of OPA1 alters the cristae periodicity and nucleoids-to-cristae proximity, leading to perturbed longitudinal nucleoid distribution within each mitochondrion. Yet, the expression of OPA1 disease-causing mutants showed some defects in cristae periodicity, but no changes in longitudinal nucleoid distribution (Fig. 5). Thus, these data allow us to dissect the role of OPA1, with fusion supporting nucleoids array amongst organelles, while cristae shaping determining intra-mitochondrial mtDNA distribution.

Different groups have studied the role of OPA1 in maintaining mtDNA and nucleoid abundance in mammalian cells [8, 33, 39, 41, 42, 53]. Yet, we emphasize the importance of describing nucleoid distribution considering the mitochondria available to contain them. Hence, we proposed a new paradigm based on our nucleoid distribution model (Fig. 1B), that incorporates mitochondrial characteristics and single-organelle analysis. Nucleoid distribution has gained attention as a functional parameter related to mtDNA distribution and inheritance [23, 54, 55]. Using this new paradigm, we showed that OPA1 plays a vital role in the distribution of the nucleoids in the mitochondrial population and at a single organelle level.

We found that in the absence of Opa1 and further rescue with OPA1 isoform 1, there is a correlation between mitochondrial morphology, cristae ultrastructure, mtDNA levels, and nucleoid number, consistent with previous reports [41]. Thus, the rescue of OPA1 isoform 1 restores mitochondrial fusion [48], morphology and cristae ultrastructure supporting the semi-regular distribution of the nucleoids and mtDNA levels, independently from the direct interaction proposed between OPA1exon 4b isoforms and mtDNA [39, 42]. Previous studies showed that rescue of the mtDNA levels upon the expression of different OPA1 isoforms in *Opa1*^−/−^ cells are related to rescue of fusion and IMM ultrastructure and not to morphology changes [41], highlighting these two components as possible regulators of mtDNA abundance.

OPA1 overexpression increased mitochondrial fusion rate [48], but the relative abundance of mtDNA molecules was unaltered. However, nucleoids are more spread within the mitochondrial population than in WT cells. This supports the idea that fusion-induced mitochondrial elongation or material exchange play a role in the distribution of nucleoids amongst mitochondria, as has been showed in the Mfn 1/2 KO model [56].

We did not find mtDNA deletions in our stable cell lines with mutant OPA1, although multiple mtDNA deletions have been found in skeletal muscle biopsies from ADOA patients [46]. The accumulation of mtDNA damage is characteristic for post-mitotic cells and it emphasizes the relevance of studying nucleoid distribution in post-mitotic tissues and on subcellular level, as the local accumulation of damaged mtDNA causes bioenergetic dysfunction in these patients [2, 18].

The nucleoid array in the mitochondrial network describes how they are spread, which is relevant for the local bioenergetic function of individual mitochondria and the cells. It is proposed that mtDNA has a “sphere of influence” over the nearby cristae, locally controlling OXPHOS function [57, 58]. Since mitochondria without nucleoids (“empty”) are accumulated in *Opa1*^−/−^ cells and WT cells expressing the ADOA-mutants, this phenotype might be related to the overall bioenergetic dysfunction found in these cells [51, 59], and most importantly, in ADOA patients [18]. In WT MEF overexpressing OPA1 and the c.2713 C > T ADOA-causing mutant, nucleoid array was altered on the organelle’s population, associated with mitochondrial shape and cristae disorganization. However, expression of the c.870+5 G > A mutant caused cristae alterations but a milder effect on the nucleoid array in the mitochondrial population, suggesting that nucleoid distribution amongst mitochondria is not exclusively directed by cristae ultrastructure.

Interestingly, intramitochondrial longitudinal distribution of nucleoids was a robust parameter only altered in *Opa1*^−/−^ cells, which also showed altered cristae periodicity and nucleoids-to-cristae proximity. Both parameters were partially rescued after OPA1 isoform 1 expression, suggesting a key role of this isoform in the arrangement of nucleoids within each mitochondrion, and the periodicity of IMM folding. This finding aligns with the super-resolution data that showed the nucleoid interspersed between the cristae in live cells [60] and the nucleoid disorganization upon cristae disorganization due to Mic60 silencing [61].

In conclusion, our new paradigm allowed us to characterize nucleoid distribution in relation to the characteristics of the mitochondria that contain them. We showed that isoform 1 of OPA1 plays a vital role in the distribution of the nucleoids in the mitochondrial population and single organelle level by means of IMM fusion and cristae organization. Furthermore, OPA1 disease-causing mutants lead to altered nucleoid distribution irrespective of the protein domain where the mutation is located, associated with fusion inhibition and cristae morphology defects, providing new insights into the pathophysiology of ADOA.

## Materials and methods

Extended Materials and Methods can be found in Supplementary Information.

### Live-cell nucleoid cluster imaging

MEF were plated on 25 mm glass coverslips and grown in culture up to 80% confluence. Nucleoids were stained using 1 µL/mL PicoGreen (15 min, 37 °C; Thermo Fisher Scientific, Massachusetts, USA) or by transfection with Tfam-GFP. Mitochondria were stained with MitoTracker Deep Red 75 nM (15 min, 37 °C; Thermo Fisher Scientific) or by transfection of mCherry-Omp25. Live-cell imaging was performed using extracellular media at 37 °C, as previously described [51]. Image acquisition was performed with 5 Z-stacks (0.19 µm interval) in a confocal microscope Zeiss LSM 880 with an Airyscan detector (63X PlanApo 1.4 NA) on the Fast SR mode, at the Advanced Microscopy Facility UMA UC. After acquisition, images were deconvolved in 3D using the ZEN Black software.

### Nucleoid cluster distribution quantification

We manually quantified nucleoid distribution and abundance inside single organelles (Fig. S1A). The manual analysis was performed in FIJI ImageJ. We detected a high Tfam-GFP intramitochondrial background noise *Opa1*^−/−^ cells, previously described as “diffused TFAM” [39]. Thus, we adjusted the B/C settings to distinguish the brightest puncta (Fig. S3A–C). To strengthen our distribution data in the mitochondrial population, we developed a semi-automated method called Mitochondrial Nucleoid Distribution (MiNuD; Fig. S1B). The input for MiNuD are the .tif files of light-microscopy images of mitochondria, nucleoids, and their respective masks obtained from FIJI threshold or Trainable Weka.

## Supplementary information

Supplemental material

Supplemental material Full Western Blot membranes

## Data Availability

The MiNuD Python package is openly available in GitHub and can be found in (https://github.com/RudgeLab/MiNuD). The data that support the findings in this paper can be found in Supplementary Materials. Any further data can be provided after reasonable request to the corresponding author.

## References

[1] Eisner V, Picard M, Hajnóczky G. Mitochondrial dynamics in adaptive and maladaptive cellular stress responses. Nat Cell Biol. 2018;20:755–65. 29950571 10.1038/s41556-018-0133-0 PMC6716149

[2] Vincent AE, Rosa HS, Pabis K, Lawless C, Chen C, Grünewald A, et al. Subcellular origin of mitochondrial DNA deletions in human skeletal muscle. Ann Neurol. 2018;84:289–301. 30014514 10.1002/ana.25288 PMC6141001

[3] Yu-Wai-Man P, Lai-Cheong J, Borthwick GM, He L, Taylor GA, Greaves LC, et al. Somatic mitochondrial DNA deletions accumulate to high levels in aging human extraocular muscles. Investig Ophthalmol Vis Sci. 2010;51:3347–53. 20164450 10.1167/iovs.09-4660 PMC2904001

[4] Brown TA, Tkachuk AN, Shtengel G, Kopek BG, Bogenhagen DF, Hess HF, et al. Superresolution fluorescence imaging of mitochondrial nucleoids reveals their spatial range, limits, and membrane interaction. Mol Cell Biol. 2011;31:4994–5010. 22006021 10.1128/MCB.05694-11 PMC3233019

[5] Kukat C, Wurm CA, Spåhr H, Falkenberg M, Larsson NG, Jakobs S. Super-resolution microscopy reveals that mammalian mitochondrial nucleoids have a uniform size and frequently contain a single copy of mtDNA. Proc Natl Acad Sci USA. 2011;108:13534–9. 21808029 10.1073/pnas.1109263108 PMC3158146

[6] Kukat C, Davies KM, Wurm CA, Spåhr H, Bonekamp NA, Kühl I, et al. Cross-strand binding of TFAM to a single mtDNA molecule forms the mitochondrial nucleoid. PNAS. 2015;112:11288–93. 26305956 10.1073/pnas.1512131112 PMC4568684

[7] Ngo H, Jt K, Dc C. The mitochondrial transcription and packaging factor Tfam imposes a U-turn on mitochondrial DNA. Nat Struct Mol Biol. 2011;18:1290–6. 22037171 10.1038/nsmb.2159 PMC3210390

[8] Chen H, Vermulst M, Wang YE, Chomyn A, Prolla TA, McCaffery JM, et al. Mitochondrial fusion is required for mtDNA stability in skeletal muscle and tolerance of mtDNA mutations. Cell. 2010;141:280–9. 20403324 10.1016/j.cell.2010.02.026 PMC2876819

[9] Ishihara T, Ban-Ishihara R, Maeda M, Matsunaga Y, Ichimura A, Kyogoku S, et al. Dynamics of mitochondrial DNA nucleoids regulated by mitochondrial fission is essential for maintenance of homogeneously active mitochondria during neonatal heart development. Mol Cell Biol. 2015;35:211–23. 25348719 10.1128/MCB.01054-14 PMC4295379

[10] Ban-Ishihara R, Ishihara T, Sasaki N, Mihara K, Ishihara N. Dynamics of nucleoid structure regulated by mitochondrial fission contributes to cristae reformation and release of cytochrome c. Proc Natl Acad Sci USA. 2013;110:11863–8. 23821750 10.1073/pnas.1301951110 PMC3718159

[11] Ota A, Ishihara T, Ishihara N. Mitochondrial nucleoid morphology and respiratory function are altered in Drp1-deficient HeLa cells. J Biochem. 2020;167:287–94. 31873747 10.1093/jb/mvz112

[12] Tezze C, Romanello V, Desbats MA, Fadini GP, Albiero M, Favaro G, et al. Age-associated loss of OPA1 in muscle impacts muscle mass, metabolic homeostasis, systemic inflammation, and epithelial senescence. Cell Metab. 2017;25:1374–1389.e6. 28552492 10.1016/j.cmet.2017.04.021 PMC5462533

[13] Renaldo F, Amati-Bonneau P, Slama A, Romana C, Forin V, Doummar D, et al. MFN2, a new gene responsible for mitochondrial DNA depletion. Brain. 2012;135:e223–e223. 22556188 10.1093/brain/aws111

[14] Rouzier C, Bannwarth S, Chaussenot A, Chevrollier A, Verschueren A, Bonello-Palot N, et al. The MFN2 gene is responsible for mitochondrial DNA instability and optic atrophy ‘plus’ phenotype. Brain. 2012;135:23–34. 22189565 10.1093/brain/awr323

[15] Bannwarth S, Ait-El-Mkadem S, Chaussenot A, Genin EC, Lacas-Gervais S, Fragaki K, et al. A mitochondrial origin for frontotemporal dementia and amyotrophic lateral sclerosis through CHCHD10 involvement. Brain. 2014;137:2329–45. 24934289 10.1093/brain/awu138 PMC4107737

[16] Genin EC, Plutino M, Bannwarth S, Villa E, Cisneros-Barroso E, Roy M, et al. CHCHD10 mutations promote loss of mitochondrial cristae junctions with impaired mitochondrial genome maintenance and inhibition of apoptosis. EMBO Mol Med. 2016;8:58–72. 26666268 10.15252/emmm.201505496 PMC4718158

[17] Vielhaber S, Debska-Vielhaber G, Peeva V, Schoeler S, Kudin AP, Minin I, et al. Mitofusin 2 mutations affect mitochondrial function by mitochondrial DNA depletion. Acta Neuropathol. 2013;125:245–56. 22926664 10.1007/s00401-012-1036-y

[18] Yu-Wai-Man P, Sitarz KS, Samuels DC, Griffiths PG, Reeve AK, Bindoff LA, et al. OPA1 mutations cause cytochrome c oxidase deficiency due to loss of wild-type mtDNA molecules. Hum Mol Genet. 2010;19:3043–52. 20484224 10.1093/hmg/ddq209 PMC2901142

[19] Sitarz KS, Almind GJ, Horvath R, Czermin B, Grønskov K, Pyle A, et al. OPA1 mutations induce mtDNA proliferation in leukocytes of patients with dominant optic atrophy. Neurology. 2012;79:1515–7. 22993284 10.1212/WNL.0b013e31826d5f60 PMC3525295

[20] Ono T, Isobe K, Nakada K, Hayashi JI. Human cells are protected from mitochondrial dysfunction by complementation of DNA products in fused mitochondria. Nat Genet. 2001;28:272–5. 11431699 10.1038/90116

[21] Iborra FJ, Kimura H, Cook PR. The functional organization of mitochondrial genomes in human cells. BMC Biol. 2004;2:9. 15157274 10.1186/1741-7007-2-9 PMC425603

[22] Legros F, Malka F, Frachon P, Lombès A, Rojo M. Organization and dynamics of human mitochondrial DNA. J Cell Sci. 2004;117:2653–62. 15138283 10.1242/jcs.01134

[23] Jajoo R, Jung Y, Huh D, Viana MP, Rafelski SM, Springer M, et al. Accurate concentration control of mitochondria and nucleoids. Science. 2016;351:169–72. 26744405 10.1126/science.aaa8714 PMC4823142

[24] Lewis SC, Uchiyama LF, Nunnari J. ER-mitochondria contacts couple mtDNA synthesis with mitochondrial division in human cells. Science. 2016;353:aaf5549. 27418514 10.1126/science.aaf5549 PMC5554545

[25] Jakobs S, Stephan T, Ilgen P, Brüser C. Light microscopy of mitochondria at the nanoscale. Annu Rev Biophys. 2020;49:289–308. 32092283 10.1146/annurev-biophys-121219-081550 PMC7610798

[26] Dlasková A, Engstová H, Špaček T, Kahancová A, Pavluch V, Smolková K, et al. 3D super-resolution microscopy reflects mitochondrial cristae alternations and mtDNA nucleoid size and distribution. Biochim Biophys Acta (BBA) - Bioenerg. 2018;1859:829–44. 10.1016/j.bbabio.2018.04.013 29727614

[27] Kopek BG, Shtengel G, Xu CS, Clayton DA, Hess HF. Correlative 3D superresolution fluorescence and electron microscopy reveal the relationship of mitochondrial nucleoids to membranes. PNAS. 2012;109:6136–41. 22474357 10.1073/pnas.1121558109 PMC3341004

[28] Blum TB, Hahn A, Meier T, Davies KM, Kühlbrandt W. Dimers of mitochondrial ATP synthase induce membrane curvature and self-assemble into rows. PNAS. 2019;116:4250–5. 30760595 10.1073/pnas.1816556116 PMC6410833

[29] Stephan T, Brüser C, Deckers M, Steyer AM, Balzarotti F, Barbot M, et al. MICOS assembly controls mitochondrial inner membrane remodeling and crista junction redistribution to mediate cristae formation. EMBO J. 2020;39:e104105. 32567732 10.15252/embj.2019104105 PMC7361284

[30] Kondadi AK, Anand R, Hänsch S, Urbach J, Zobel T, Wolf DM, et al. Cristae undergo continuous cycles of membrane remodelling in a MICOS-dependent manner. EMBO Rep. 2020;21:e49776. 32067344 10.15252/embr.201949776 PMC7054676

[31] Quintana-Cabrera R, Quirin C, Glytsou C, Corrado M, Urbani A, Pellattiero A, et al. The cristae modulator Optic atrophy 1 requires mitochondrial ATP synthase oligomers to safeguard mitochondrial function. Nat Commun. 2018;9:3399. 30143614 10.1038/s41467-018-05655-x PMC6109181

[32] Frezza, Cipolat C, Brito S, de OM, Micaroni M, Beznoussenko GV, et al. OPA1 controls apoptotic cristae remodeling independently from mitochondrial fusion. Cell. 2006;126:177–89. 16839885 10.1016/j.cell.2006.06.025

[33] Fry MY, Navarro PP, Hakim P, Ananda VY, Qin X, Landoni JC, et al. In situ architecture of Opa1-dependent mitochondrial cristae remodeling. EMBO J. 2024;43:391–413. 38225406 10.1038/s44318-024-00027-2 PMC10897290

[34] Song Z, Ghochani M, McCaffery JM, Frey TG, Chan DC. Mitofusins and OPA1 mediate sequential steps in mitochondrial membrane fusion. Mol Biol Cell. 2009;20:3525–32. 19477917 10.1091/mbc.E09-03-0252 PMC2719570

[35] Liu X, Weaver D, Shirihai O, Hajnóczky G. Mitochondrial ‘kiss-and-run’: interplay between mitochondrial motility and fusion–fission dynamics. EMBO J. 2009;28:3074–89. 19745815 10.1038/emboj.2009.255 PMC2771091

[36] Anand R, Wai T, Baker MJ, Kladt N, Schauss AC, Rugarli E, et al. The i-AAA protease YME1L and OMA1 cleave OPA1 to balance mitochondrial fusion and fission. J Cell Biol. 2014;204:919–29. 24616225 10.1083/jcb.201308006 PMC3998800

[37] Herlan M, Vogel F, Bornhovd C, Neupert W, Reichert AS. Processing of Mgm1 by the rhomboid-type protease Pcp1 is required for maintenance of mitochondrial morphology and of mitochondrial DNA. J Biol Chem. 2003;278:27781–8. 12707284 10.1074/jbc.M211311200

[38] Sesaki H, Southard SM, Yaffe MP, Jensen RE. Mgm1p, a Dynamin-related GTPase, is essential for fusion of the mitochondrial outer membrane. MBoC. 2003;14:2342–56. 12808034 10.1091/mbc.E02-12-0788 PMC194884

[39] Yang L, Tang H, Lin X, Wu Y, Zeng S, Pan Y, et al. OPA1-Exon4b binds to mtDNA D-loop for transcriptional and metabolic modulation, independent of mitochondrial fusion. Front Cell Dev Biol. 2020;8:180. 32373606 10.3389/fcell.2020.00180 PMC7179665

[40] Patten DA, Wong J, Khacho M, Soubannier V, Mailloux RJ, Pilon-Larose K, et al. OPA1-dependent cristae modulation is essential for cellular adaptation to metabolic demand. EMBO J. 2014;33:2676–91. 25298396 10.15252/embj.201488349 PMC4282575

[41] Del Dotto V, Mishra P, Vidoni S, Fogazza M, Maresca A, Caporali L, et al. OPA1 isoforms in the hierarchical organization of mitochondrial functions. Cell Rep. 2017;19:2557–71. 28636943 10.1016/j.celrep.2017.05.073

[42] Elachouri G, Vidoni S, Zanna C, Pattyn A, Boukhaddaoui H, Gaget K, et al. OPA1 links human mitochondrial genome maintenance to mtDNA replication and distribution. Genome Res. 2011;21:12–20. 20974897 10.1101/gr.108696.110 PMC3012919

[43] Lenaers G, Hamel C, Delettre C, Amati-Bonneau P, Procaccio V, Bonneau D, et al. Dominant optic atrophy. Orphanet J Rare Dis. 2012;7:46. 22776096 10.1186/1750-1172-7-46 PMC3526509

[44] Yu-Wai-Man P, Trenell MI, Hollingsworth KG, Griffiths PG, Chinnery PF. OPA1 mutations impair mitochondrial function in both pure and complicated dominant optic atrophy. Brain. 2011;134:e164–e164. 20952381 10.1093/brain/awq288 PMC3069699

[45] Yu-Wai-Man P, Griffiths PG, Gorman GS, Lourenco CM, Wright AF, Auer-Grumbach M, et al. Multi-system neurological disease is common in patients with OPA1 mutations. Brain. 2010;133:771–86. 20157015 10.1093/brain/awq007 PMC2842512

[46] Amati-Bonneau P, Valentino ML, Reynier P, Gallardo ME, Bornstein B, Boissière A, et al. OPA1 mutations induce mitochondrial DNA instability and optic atrophy ‘plus’ phenotypes. Brain. 2008;131:338–51. 18158317 10.1093/brain/awm298

[47] Hudson G, Amati-Bonneau P, Blakely EL, Stewart JD, He L, Schaefer AM, et al. Mutation of OPA1 causes dominant optic atrophy with external ophthalmoplegia, ataxia, deafness and multiple mitochondrial DNA deletions: a novel disorder of mtDNA maintenance. Brain. 2008;131:329–37. 18065439 10.1093/brain/awm272

[48] Cartes-Saavedra B, Lagos D, Macuada J, Arancibia D, Burté F, Sjöberg-Herrera MK, et al. OPA1 disease-causing mutants have domain-specific effects on mitochondrial ultrastructure and fusion. Proc Natl Acad Sci USA. 2023;120:e2207471120. 36927155 10.1073/pnas.2207471120 PMC10041121

[49] Pastukh V, Shokolenko I, Wang B, Wilson G, Alexeyev M. Human mitochondrial transcription factor A possesses multiple subcellular targeting signals. FEBS J. 2007;274:6488–99. 18028422 10.1111/j.1742-4658.2007.06167.x

[50] Huff J. The Fast mode for ZEISS LSM 880 with Airyscan: high-speed confocal imaging with super-resolution and improved signal-to-noise ratio. Nat Methods. 2016;13:i–ii.

[51] Cartes-Saavedra B, Macuada J, Lagos D, Arancibia D, Andrés ME, Yu-Wai-Man P, et al. OPA1 modulates mitochondrial Ca^2+^ uptake through ER-Mitochondria coupling. Front Cell Dev Biol. 2022;9:774108. 35047497 10.3389/fcell.2021.774108 PMC8762365

[52] Agier V, Oliviero P, Lainé J, L’Hermitte-Stead C, Girard S, Fillaut S, et al. Defective mitochondrial fusion, altered respiratory function, and distorted cristae structure in skin fibroblasts with heterozygous OPA1 mutations. Biochim Biophys Acta (BBA) Mol Basis Dis. 2012;1822:1570–80. 10.1016/j.bbadis.2012.07.002 22800932

[53] Rodríguez-Nuevo A, Díaz-Ramos A, Noguera E, Díaz-Sáez F, Duran X, Muñoz JP, et al. Mitochondrial DNA and TLR9 drive muscle inflammation upon Opa1 deficiency. EMBO J. 2018;37:e96553. 29632021 10.15252/embj.201796553 PMC5978453

[54] Tauber J, Dlasková A, Šantorová J, Smolková K, Alán L, Špaček T, et al. Distribution of mitochondrial nucleoids upon mitochondrial network fragmentation and network reintegration in HEPG2 cells. Int J Biochem Cell Biol. 2013;45:593–603. 23220174 10.1016/j.biocel.2012.11.019

[55] Ilamathi HS, Ouellet M, Sabouny R, Desrochers-Goyette J, Lines MA, Pfeffer G, et al. A new automated tool to quantify nucleoid distribution within mitochondrial networks. Sci Rep. 2021;11:22755. 34815439 10.1038/s41598-021-01987-9 PMC8610998

[56] Silva Ramos E, Motori E, Brüser C, Kühl I, Yeroslaviz A, Ruzzenente B, et al. Mitochondrial fusion is required for regulation of mitochondrial DNA replication. Barsh GS, editor. PLoS Genet. 2019;15:e1008085. 10.1371/journal.pgen.1008085 PMC6553695 31170154

[57] Busch KB, Kowald A, Spelbrink JN. Quality matters: how does mitochondrial network dynamics and quality control impact on mtDNA integrity? Philos Trans R Soc B Biol Sci. 2014;369:20130442. 10.1098/rstb.2013.0442 PMC4032518 24864312

[58] Jakubke C, Roussou R, Maiser A, Schug C, Thoma F, Bunk D, et al. Cristae-dependent quality control of the mitochondrial genome. Sci Adv. 2021;7:eabi8886. 34516914 10.1126/sciadv.abi8886 PMC8442932

[59] Olichon A, Landes T, Arnauné‐Pelloquin L, Emorine LJ, Mils V, Guichet A, et al. Effects of OPA1 mutations on mitochondrial morphology and apoptosis: Relevance to ADOA pathogenesis. J Cell Physiol. 2007;211:423–30. 17167772 10.1002/jcp.20950

[60] Liu T, Stephan T, Chen P, Keller-Findeisen J, Chen J, Riedel D, et al. Multi-color live-cell STED nanoscopy of mitochondria with a gentle inner membrane stain. Proc Natl Acad Sci USA. 2022;119:e2215799119. 36534799 10.1073/pnas.2215799119 PMC9907107

[61] Li H, Ruan Y, Zhang K, Jian F, Hu C, Miao L, et al. Mic60/Mitofilin determines MICOS assembly essential for mitochondrial dynamics and mtDNA nucleoid organization. Cell Death Differ. 2016;23:380–92. 26250910 10.1038/cdd.2015.102 PMC5072434

