# Supplementary material for: OPA1 and disease-causing mutants perturb mitochondrial nucleoid distribution
Source: Cell Death Dis. 2024 Nov 30;15(11):870. doi: 10.1038/s41419-024-07165-9 (PMC11608329; doi:10.1038/s41419-024-07165-9)

FULL WESTERN BLOT MEMBRANES

Here are the two replicates of the Western blot experiment in **Fig. S1.**

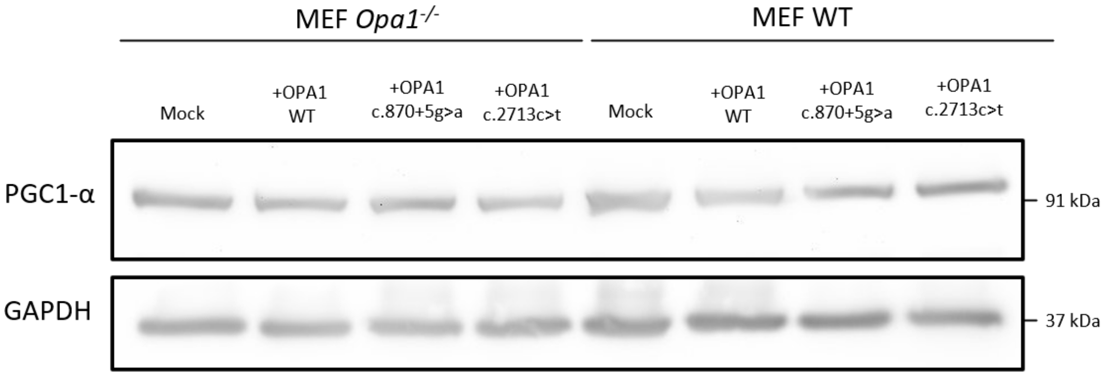

Full membrane - PGC1-alfa

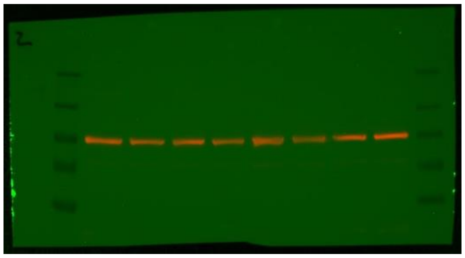

Full membrane -GAPDH

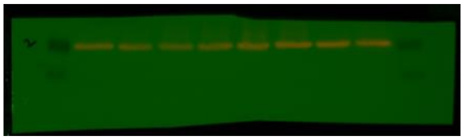

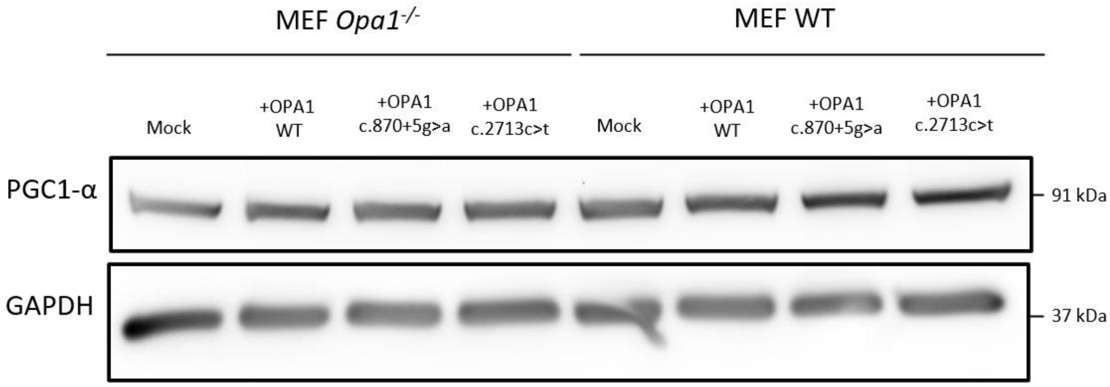

Full membrane - PGC1-alfa

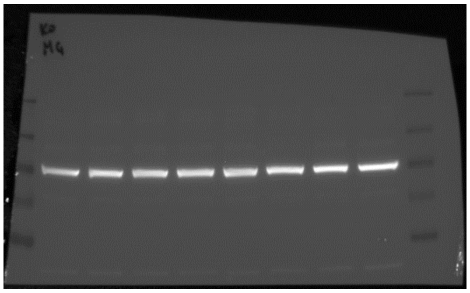

Full membrane -GAPDH

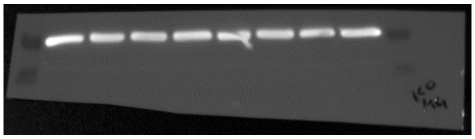

Supplement: Supplementary file 2 — Supplemental material Full Western Blot membranes [file 41419_2024_7165_MOESM2_ESM.pdf]
